# Association of energy availability with resting metabolic rates in competitive female teenage runners: a cross-sectional study

**DOI:** 10.1186/s12970-021-00466-w

**Published:** 2021-11-16

**Authors:** Norimitsu Kinoshita, Eriko Uchiyama, Kazuko Ishikawa-Takata, Yuka Yamada, Kenta Okuyama

**Affiliations:** 1 Faculty of Sports and Health Studies, Hosei University, 4342 Aihara, Tokyo 194-0298 Machida, Japan; 2 Health and Nutrition, National Institutes of Biomedical Innovation, 1-23-1 Toyama, Tokyo 162-8636 Shinjuku, Japan; 3 Faculty of Applied Bioscience, Tokyo University of Agriculture, 1-1-1 Sakuragaoka, Tokyo 156-8502 Setagaya, Japan; 4 Center for Primary Health Care Research, Department of Clinical Sciences Malmö, Lund University, Jan Waldenströms Gata 35, 20502 Malmö, Sweden

**Keywords:** Whole room calorimeter, Adaptive thermogenesis, Female athlete triad, Adolescence, Dual-energy X-ray absorptiometry, Long-distance running

## Abstract

**Background:**

Resting metabolic rate (RMR) has been examined as a proxy for low energy availability (EA). Previous studies have been limited to adult athletes, despite the serious health consequences of low EA, particularly during adolescence. This study aimed to explore the relationship between RMR and EA in competitive teenage girl runners.

**Methods:**

Eighteen girl runners (mean ± standard-deviation; age, 16.8 ± 0.9 years; body mass, 45.6 ± 5.2 kg, %fat, 13.5 ± 4.2 %) in the same competitive high-school team were evaluated. Each runner was asked to report dietary records with photos and training logs for seven days. Energy intake (EI) was assessed by Registered Dietitian Nutritionists. The runners were evaluated on a treadmill with an indirect calorimeter to yield individual prediction equations for oxygen consumption using running velocity and heart rate (HR). Exercise energy expenditure (EEE) was calculated by the equations based on training logs and HR. Daily EA was calculated by subtracting EEE from EI. The daily means of these variables were calculated. RMR was measured early in the morning by whole-room calorimetry after overnight sleep on concluding the final day of the seven-day assessment. The ratio of measured RMR to predicted RMR (RMR ratio) was calculated by race, age, sex-specific formulae, and Cunningham’s equation. Body composition was measured using dual-energy X-ray absorptiometry. Bivariate correlation analyses were used to examine the relationship between variables.

**Results:**

RMR, EI, EEE, and EA were 26.9 ± 2.4, 56.8 ± 15.2, 21.7 ± 5.9, and 35.0 ± 15.0 kcal⋅kg^−1^ FFM⋅d^−1^, respectively. RMR reduced linearly with statistical significance, while EA decreased to a threshold level (30 kcal⋅kg^−1^ FFM⋅d^−1^) (*r=* 0.58, *p=* 0.048). Further reduction in RMR was not observed when EA fell below the threshold. There was no significant correlation between RMR ratios and EA, irrespective of the prediction formulae used.

**Conclusions:**

These results suggest that RMR does not reduce with a decrease in EA among highly competitive and lean teenage girl runners. RMR remains disproportionally higher than expected in low EA states. Free-living teenage girl runners with low EA should be cautiously identified using RMR as a proxy for EA change.

## Background

Energy availability (EA) is defined as the energy obtained from dietary intake after subtracting the energy consumed by exercise training [1]. EA may represent energy that is spared for the body’s essential functions, such as reproduction or bone formation. When EA is substantially decreased by the restriction of energy intake (EI), an increase in exercise energy expenditure (EEE), or both, these functions are sacrificed [1]. In fact, cause-effect relationships between low EA and reproductive hormone disturbances or bone metabolism have been demonstrated [2–4]. These disturbances are manifested as amenorrhea [5] and low bone mass [6] in female athletes and are considered as interrelated components of the female athlete triad [1].

Since it is difficult to accurately measure EA in practice, reductions in resting metabolic rate (RMR) have been examined as a proxy for low EA [7–9]. Since severe caloric restriction reduces the RMR [10], it is reasonable to expect the same metabolic adaptation to occur in low EA states. However, only a few studies have directly examined the association between low EA and a reduction in RMR among female athletes [7, 8, 11]. Similarly, RMR is influenced by exercise training, which may conversely preserve RMR in negative energy balance or weight-loss programs by restricting EI [12, 13]. Since EEE is needed to determine EA, further research is required prior to using reduced RMR to identify athletes with low EA.

Long-distance running is a weight bearing endurance sport wherein low body fat helps in enhancing performance. Accordingly, competitive distance runners likely control their EI [14], raising concern that many suffer from low EA [15, 16]. Low EA could be an important factor in adolescent female athletes, affecting lifelong bone health since adolescence is a critical period of bone mineral accrual [17] where the greatest gains are attained. Thinness, linked to insufficient EI [15, 16], which is related to reduced RMR [10, 15] or low EA [15, 18], is prevalent among adolescent female runners [19, 20]. Therefore, this age group of runners is likely to have low EA with suppressed RMR. However, studies regarding EA measured in adolescent female runners in relation to RMR have not been performed. Further, research of EA in Asian populations is limited. Consistency among different races is important to strengthen causal inference in the influence of low EA on metabolic adaptation [21]. Furthermore, evidence from free-living athletes is relevant to sports medicine practice. Therefore, this study aimed to explore the association between EA and RMR in free-living Japanese competitive girl runners. Since RMR is influenced by race, age, sex [22], and the method of measurement [23], we examined a small group of runners from the same team with highly homogeneous backgrounds and measured RMR according to the rigorous testing procedure for basal metabolic rate [23] using a whole room calorimeter.

## Methods

### Participants

Eighteen female teenage runners (15−19 years old) from the same high school team were evaluated from 2015 to 2020. One runner had graduated at the time of measurement but continued to train under the instruction of the same team coach. All participants were Japanese, competitive middle- or long-distance (800−5000 m) runners, with a personal best record of 4 min 43 s ± 14 s and 9 min 58 s ± 31 s for 1500 m and 3000 m races, respectively. During the five-year study period, the team consistently ranked high, won the Prefectural Championship Road Running Relay race three times, and advanced to the relevant national championship. Runners from high schools representing the 47 prefectures of Japan participate in the national championship, and they are considered as the best high school runners. The team had been ranked average in the race.

All physical training was organized and supervised by the same coach during the study period, while the diet was left to the discretion of each runner. Some participants lived in the same dormitory; in which case their daily lifestyles were strictly controlled. Training sessions were usually performed twice a day: early in the morning and in the evening. Training comprised jogging, tempo running, and high-intensity interval training. Resistance and agility training was similarly performed almost daily.

Training details and diet were regularly recorded by the athletes in their diaries. The maximal running mileage was approximately 100 km per week, and the runners went on intensive training camps for up to three weeks, four to five times a year, where the training intensity, daily training hours, and mileage were substantially increased. No significant abnormalities were observed in the participants following mandatory medical check-ups performed in the high school.

### Study protocol

The study was conducted without interrupting school events, classes, or scheduled training programs, and without modifying the diet of the participants. Particularly, the days requiring an overnight stay at the laboratory were carefully chosen, so as not to affect daily exercise training. Consequently, the day of RMR measurement was set for a previously planned rest day, free from exercise training, which was mostly Sunday, and the last exercise training was scheduled on the morning of the day before the RMR measurement. This was done to secure at least 17 h of abstinence from exercise training for the measurement of RMR. This met the American Dietetic Association’s recommendation on best practice methods to measure RMR [24].

**Fig. 1:**
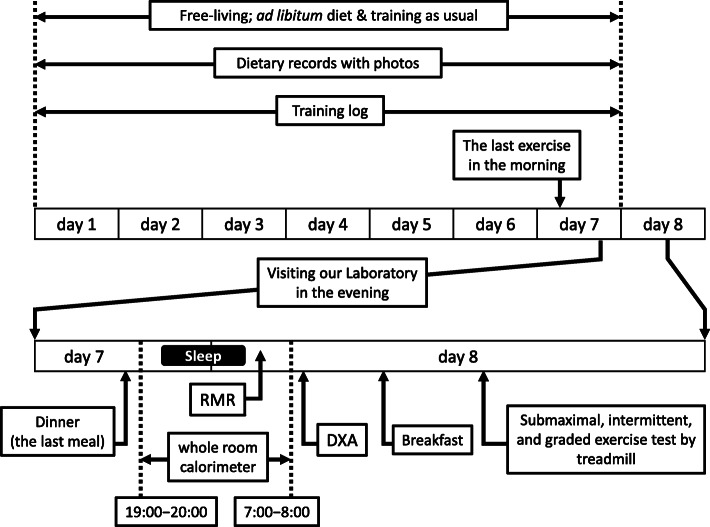
Study protocol. RMR: resting metabolic rate; DXA: dual-energy X-ray absorptiometry

The study was scheduled for a total of eight consecutive days (Fig. 1), and the participants were asked to spend their time as usual without changing exercise training and diet. The diets of the participants were recorded with training logs for seven days, and the participants visited our laboratory on the evening of the seventh day after refraining from training since noon. The time of dinner was unchanged and served as desired. After dinner, the participant spent the night in the whole room calorimeter of the laboratory, and RMR was measured early the next morning. The time of sleeping and waking was unchanged for each individual. Immediately after RMR measurement, body composition was evaluated by dual-energy X-ray absorptiometry (DXA), and a submaximal exercise test was performed on a treadmill using an indirect calorimeter.

### EI

Runners were instructed to record every meal, including drinks and nutritional supplements, with photographs, and report the meals to Registered Dietitian Nutritionists (K.I.T. and Y.Y.) via daily e-mails, which included details regarding the type of food, ingredients, seasoning, and preparation. These details were obtained from and confirmed by the participants’ parents who served the meals. A ruler supplied by the nutritionists was incorporated into each photo, taken before and after each meal, to estimate the volume of food consumed. As the participants consumed steamed rice with almost every meal, the weight of the rice was measured each time using a digital scale (KD-192, TANITA, Tokyo, Japan).

On the seventh or eighth day of the study, the nutritionists conducted face-to-face interviews with each subject to confirm the accuracy of all records. Daily EI was calculated using the Standard Tables of Food Composition in Japan - 2015 (Seventh Revised Version) [25]. The mean EI for all seven days was calculated and used for analysis.

### EEE

EEE was calculated for running and other types of training (resistance training and agility drills) separately, using individual prediction equations for the total oxygen consumption (VO_2_) of each runner. We used running speed to estimate EEE for the running training, whereas heart rate (HR) was used for resistance training and agility drills. A timekeeper checked and recorded lap times (each 200 m for track training and, at most, 1 km for road running) with a stopwatch for each subject. Similarly, the participants recorded their own lap times with a sports wristwatch and attempted to run at the speed set by the coach. Each lap time was recorded in the training logs, and the running speed was calculated by the lap time for each distance. HR was recorded every 5 s by a wearable HR monitor (Polar Loop, Polar Electro Oy, Kempele, Finland) and uploaded to the POLAR FLOW website [26]. The HR data was exported in .csv format and was analyzed.

The runners were assessed on a treadmill at 1 % slope with breath-by-breath indirect calorimetry (Quark CPET, COSMED, Rome, Italy) to yield submaximal steady-state VO_2_ (ml⋅kg^−1^⋅min^−1^) for three or four sets at 10, 12, 14, and 15 km⋅hr^−1^. The duration of each stage was set at 5 min and separated with more than 5 min rest. The submaximal steady-state VO_2_ was plotted according to the velocity and HR, and equations to estimate VO_2_ were determined using linear regression analysis. The mean R^2^ of the regression lines was 0.986 (0.920−1.000) and 0.992 (0.967−1.000), respectively.

The participants reported that they had occasionally engaged in sports activities, such as volleyball or swimming, apart from those in their regular training program. Although the amount of energy expenditure in these activities was relatively minor compared to regular training, we included them in calculating total EEE, using a youth compendium of physical activities [27]. Finally, total EEE (kcal) was calculated using the sum of training and other activities with a caloric equivalent of 5 kcal⋅L^−1^ of O_2_ and subtracting RMR (kcal) of the equivalent duration of exercise. The mean EEE for all seven days was analyzed.

### EA

Daily EA was calculated by subtracting EEE from EI and adjusting for fat free mass (FFM). The means of the seven days were calculated and used for analysis. As the EI on day 1 for one subject, and on days 1 and 2 for another subject, were not available, we calculated these means for six and five days, respectively, for EA as well as EI and EEE.

EA below the threshold value of 30 kcal⋅kg^−1^ FFM⋅d^−1^ was defined as low EA according to the American College of Sports Medicine [1]. Similarly, we used a cut-off value of 45 kcal⋅kg^−1^ FFM⋅d^−1^ to identify optimal EA [1]. Using t-tests, we compared the means of the values above and below the threshold of 30 (non-low EA vs. low EA, respectively) as well as above and below the cut-off value of 45 (optimal EA vs. non-optimal EA, respectively) for each continuous variable.

### RMR

RMR was measured using a whole room calorimeter (Fuji Medical Science, Chiba, Japan) early in the morning after overnight sleep. The room measured 3.7 (W) × 2.7 (D) × 2.5 (H) m with an internal volume of 25.2 m^3^. The room had a bed with a thick and comfortable mattress (Sealy Corporation, Trinity, NC, USA), a neck pillow made of a pressure-relieving material (Tempur-Pedic International, Inc., Lexington, KY, USA), a TV set, washbasin, and a toilet. The airflow was kept at 50 L⋅min^−1^ with temperature and relative humidity set to 25.0° C and 50.0 %, respectively. The air was analyzed by mass spectrometry (Thermo Scientific ^TM^ Prima PRO Process Mass Spectrometer, Thermo Fisher Scientific, Cheshire, UK). Calibration was performed periodically. VO_2_ and carbon dioxide production were calculated according to Henning et al. [28], and energy expenditure (kcal) was estimated by Weir’s equation [29]. The accuracy was verified by an alcohol combustion test before each experiment. Test-retest reliability for energy consumption at rest was calculated for five volunteers, and the coefficients of variation (CV) were 4.4 ± 1.3 %.

The participants were familiarized with the procedure by visiting our laboratory and observing the facility prior to the study to reduce emotional stress. They entered the room after finishing dinner (18:00 − 20:00) the day before RMR measurement (Fig. 1). The participants were instructed to stay quietly in the room without any exercise until they went to bed as per their routine (21:00 − 23:00) and stay motionless when they woke up the next morning.

The time of waking was determined beforehand (05:00 − 06:00) as per their routine, whereupon calm, classical music (Mozart: Klarinettenkonzert KV 622; Adagio) was played through a wireless communication device (TY-WSA10, Toshiba Corporation, Tokyo, Japan) for 3 min, and the room was dimly lit using indirect lighting. After confirming that the subject was fully awake using the communication device, they were asked to keep calm and stay motionless. If necessary, they could turn over and stretch gently, lying on the bed for a few minutes, or use the toilet, before RMR measurement.

For RMR measurement, the participants were instructed not to move on the bed and remain supine for 40 to 60 min (50 ± 12 min). The time was announced every 5 min in a gentle and quiet voice during the measurement using the wireless device. We ensured that the participants remembered every announcement after finishing the RMR measurement and that they remained awake during measurement.

**Fig. 2:**
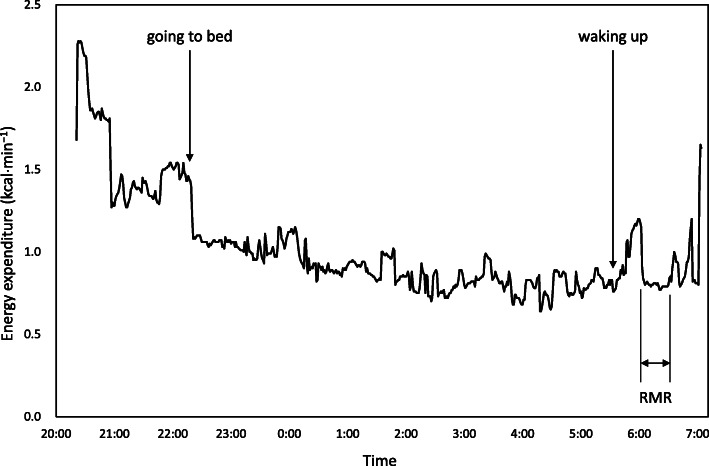
Energy expenditure measured in a whole room calorimeter. RMR: resting metabolic rate

The steady-state of calculated energy expenditure was visually assessed by a PC monitor after obtaining approximately 20 min of data, a duration which was adopted to determine RMR (Fig. 2). The duration for the calculation was 21 ± 4 min, and CV of energy expenditure for the duration were 3.7 ± 1.9 %. RMR was measured between 05:00 − 07:00, after 7.7 ± 0.7 h of sleep, 17−19 h since the last training session, and after abstaining from food for 10.7 ± 0.8 h. A wrist-worn accelerometer (wGT3X-BT, Actigraph, Pensacola, FL, USA) was mounted on the wrist of the participants, and the data was analyzed using ActiLife 6.11.6 software (ActiGraph, Pensacola, FL, USA), retrospectively, to confirm that the participants had not moved during RMR measurement. The ratio of measured RMR to predicted RMR (RMR ratio) was calculated. Considering the influence of race, age, and sex on RMR, we used the following formulae to determine a predicted RMR (kcal⋅d^−1^) for Japanese females (J1): < 18 years old (*n* = 15), 7.64 × body mass (kg) + 4.22 × height (cm) – 22.5 × age (years) + 526 [30]; ≥ 18 years old (*n* = 3), (0.0481 × body mass (kg) + 0.0234 × height (cm) – 0.0138 × age (years) – 0.9708) × 1000/4.186 [31]. Since accounting for FFM is supposedly appropriate for RMR prediction in athletes [9], we used Cunningham’s equation (C): 500 + 22 × FFM (kg) [32], and the equation developed for adult female Japanese athletes (J2): 36 + 26.9 × FFM (kg) [33] to predict RMR.

### Anthropometry and body composition

Height and body mass were measured using an automatic digital scale (AD-6228, A&D, Tokyo, Japan). Areal bone mineral density (BMD) of total body less head (BMD_TBLH_) (g⋅cm^−2^), with Z-score and body composition, was measured by DXA (Prodigy, GE healthcare, Madison, WI, USA), early in the morning directly after RMR measurement. A quality assurance test was performed before each scan using a phantom provided by the manufacturer. We assessed test-retest reliability using 10 girl runners from the same team as the participants. The CV for BMD, fat mass, and lean soft tissue mass were 0.4 %, 1.8 %, and 0.3 %, respectively. The same investigator (N.K.) conducted all scans, and enCORE software versions 13 and 16 (GE Healthcare, Madison, WI, USA) were used for data processing. Body mass index (BMI) was calculated as body mass (kg) divided by height squared (m^2^). FFM was calculated by adding bone mineral content to lean soft tissue mass.

### Menstrual history

Menstrual history was investigated using a questionnaire alongside a personal interview. If the frequency of menstruation in the last 12 months was ≤ 3, or the three most recent consecutive menstrual cycles were absent, it was classified as amenorrhea [34]. If the menstruation frequency in the last 12 months was three times, but the menstruation mostly occurred over the recent 3 months, we regarded it as resumed menstruation and did not classify it as amenorrhea.

### Statistical analysis

All statistical analyses were conducted under the guidance of a statistical expert (K.O.) with SPSS^®^ version 25.0 J for Windows (IBM Japan, Ltd., Tokyo, Japan). T-tests were performed to define the differences between two EA groups with Levene’s test for equality of variances. The Fisher’s exact test was used to assess the difference in frequency of amenorrhea between the two groups. Pearson’s correlation analysis was used to evaluate the association between two continuous variates. We primarily tested the hypothesis that RMR reduces linearly with a decrease in EA. Further, the relationship was evaluated for each group of the EA level. A P value < 0.05 was considered statistically significant. Given the exploratory nature and the small number of study participants, effect sizes were reported as Hedges’ *g* with a correction for small sample bias for t-tests [35] and ϕ for comparing the frequency of amenorrhea. Effect sizes were calculated using R version 4.0.3, and values were presented as mean ± standard deviation.

## Results

The mean daily EI and EEE of the participants ranged from 1236 to 3016 and 441 to 1285 kcal⋅d^−1^, respectively. EA varied widely from 6.5−55.4 kcal⋅kg^−1^ FFM⋅d^−1^, and six of the 18 participants were categorized into the low EA group. The participants were exclusively thin and lean (Table 1); however, body mass, BMI, fat mass, FFM, BMD_TBLH_, and Z-score were significantly higher in participants with low EA than those above the threshold (30 kcal⋅kg^−1^ FFM⋅d^−1^). Similarly, the low EA group had a higher percentage of body fat, but without significant difference from their counterparts. The running performances in participants with EA above the threshold were better than those with low EA. However, these were not significantly different; the personal best records of 1500 and 3000 m races were 4 min 40 s ± 15 s and 9 min 55 s ± 34 s in participants with EA above the threshold, and 4 min 49 s ± 12 s and 10 min 4 s ± 27 s in those with low EA (*p* = 0.24 and 0.56, *g* = 0.59 and 0.28, respectively).

**Table 1:** Comparison of anthropometric characteristics between groups according to energy availability level

		EA (kcal⋅kg^−1^ FFM⋅d^−1^)			
	All	> 30	< 30	*p*	Effect size
N	18	12	6		
Age (years)	16.8 ± 0.9	16.8 ± 0.9	16.8 ± 0.8	>0.99	<0.01
Height (cm)	160.3 ± 5.8	159.2 ± 6.5	162.6 ± 3.3	0.261	0.56
Body mass (kg)	45.6 ± 5.2	43 ± 3.6	51 ± 3.4	<0.001	2.14
BMI (kg⋅m^−2^)	17.7 ± 1.3	16.9 ± 0.5	19.3 ± 1.0	0.001	3.22
% fat (%)	13.5 ± 4.2	12.0 ± 2.6	16.6 ± 5.2	0.084	1.21
Fat mass (kg)	6.3 ± 2.6	5.2 ± 1.3	8.6 ± 3.2	0.047	1.56
Fat free mass (kg)	39.3 ± 3.5	37.8 ± 3.2	42.4 ± 1.4	0.005	1.56
BMD_TBLH_ (g⋅cm^−2^)	1.021 ± 0.039	1.006 ± 0.037	1.049 ± 0.027	0.024	1.18
Z-score (TBLH)	0.1 ± 0.5	−0.1 ± 0.5	0.4 ± 0.3	0.042	1.05
Amenorrhea (%)	50	41.7	66.7	0.620	0.24

Values are presented as mean ± standard deviation

EA: energy availability, BMI: body mass index, BMD_TBLH_: bone mineral density of total body less head

Half of the participants (*n* = 9) were amenorrheic; however, there was no significant difference in the frequencies of amenorrhea between those two groups (Table 1). There were two runners of resumed menstruation in the group of EA above 30 kcal⋅kg^−1^ FFM⋅d^−1^. The rest of the participants’ periods were regular, with a mean frequency of 29.6 ± 0.9 days throughout the last 12 months. RMR, EI, and EEE were not significantly different between participants with or without amenorrhea (27.0 vs. 26.8, 54.4 vs. 59.1, and 23.4 vs. 19.9 kcal⋅kg^−1^ FFM⋅d^−1^, all *p* > 0.05, *g* = 0.08, 0.29, and 0.56, respectively). EA was larger in participants without amenorrhea than in those with amenorrhea; however, it was not statistically significant (39.0 vs. 31.0 kcal⋅kg^−1^ FFM⋅d^−1^, *p* = 0.276, *g* = 0.51).

The participants with low EA consumed significantly lower EI than those above the threshold, whereas EEE values were not significantly different between the two groups (Table 2). RMR was not different between EA above vs. below 30 kcal⋅kg^−1^ FFM⋅d^−1^ (Table 3). When a comparison was performed by setting the EA cut-off value at 45 kcal⋅kg^−1^ FFM⋅d^−1^, above which EA was regarded as optimal [1], RMR (kcal⋅kg^−1^ FFM⋅d^−1^) was significantly higher in participants with optimal EA than in those with non-optimal EA (*p* = 0.016, *g* = 1.28). Measured RMRs were lower than predicted RMRs, regardless of EA level, and there were no significant differences in RMR ratios between the participants with low EA and those above the threshold, irrespective of the prediction formulae used (Table 3).

**Table 2:** Comparison of energy intake and exercise energy expenditure between groups according to energy availability level

		EA (kcal⋅kg^−1^ FFM⋅d^−1^)			
	All	> 30	< 30	*p*	Effect size
N	18	12	6		
Energy intake					
(kcal⋅kg^−1^ FFM⋅d^−1^)	56.8 ± 15.2	65.4 ± 10.0	39.5 ± 6.2	<0.001	2.75
Exercise energy expenditure					
(kcal⋅kg^−1^ FFM⋅d^−1^)	21.7 ± 5.9	21.0 ± 5.9	23.1 ± 6.3	0.502	0.33
Energy availability					
(kcal⋅kg^−1^ FFM⋅d^−1^)	35.0 ± 15.0	44.3 ± 6.6	16.5 ± 7.5	<0.001	3.85

Values are presented as mean ± standard deviation

EA: energy availability, FFM: fat free mass (kg)

**Table 3:** Comparison of resting metabolic rate and ratio between groups according to energy availability level

		EA (kcal⋅kg^−1^ FFM⋅d^−1^)			
	All	> 30	< 30	*p*	Effect size
N	18	12	6		
RMR					
(kcal⋅kg^−1^ FFM⋅d^−1^)	26.9 ± 2.4	27.4 ± 2.6	25.9 ± 1.7	0.231	0.59
RMR ratio					
J1	0.90 ± 0.08	0.90 ± 0.09	0.90 ± 0.07	0.969	0.02
J2	0.96 ± 0.08	0.98 ± 0.08	0.93 ± 0.06	0.288	0.52
C	0.77 ± 0.06	0.77 ± 0.07	0.77 ± 0.04	0.878	0.05

Values are presented as mean ± standard deviation

EA: energy availability, RMR: resting metabolic rate, RMR ratio: measured/predicted RMR, FFM: fat free mass (kg)

Predicted RMR was calculated by race, age, and sex specific formulae [30, 31] in J1; formula developed for Japanese adult female athletes accounting for FFM [33] in J2; Cunningham’s equation [32] in C

**Fig. 3:**
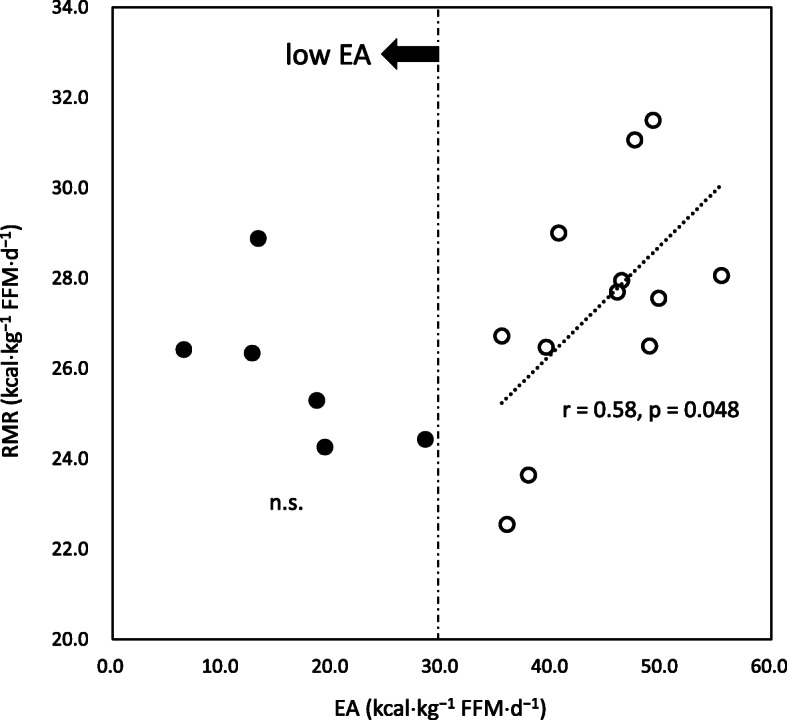
Association between RMR and energy availability. EA: energy availability; RMR: resting metabolic rate; FFM: fat free mass

Pearson’s correlation analyses demonstrated that RMR was significantly correlated with EI (*r* = 0.48, *p* = 0.043). However, RMR was not significantly correlated with EEE and EA (*r* = 0.28 and 0.38, *p* = 0.261 and 0.120, respectively). The association between RMR and EA appeared to be non-linear (Fig. 3). RMR reduced as EA decreased up to a level of 30 kcal⋅kg^−1^ FFM⋅d^−1^. There was a significant linear correlation between RMR and EA among the participants with EA above 30 kcal⋅kg^−1^ FFM⋅d^−1^. This was not the case in the low EA group. There were no significant correlations between RMR ratios and EA, irrespective of the prediction formulae used (J1, *r *< 0.01; J2, *r* = 0.34; C, *r* = 0.12, all *p* > 0.05).

## Discussion

This is the first study to report RMR in competitive teenage girl runners in relation to EA. The homogeneity of well-defined participants is one of the strengths of this study. Participants were exclusively Japanese, highly competitive runners in their late teens, who belonged to the same team, with the training regimen controlled by a single coach. Accordingly, the modes of exercise were uniform, and EEE was not controlled by the participants themselves. Conversely, we allowed the participants *ad libitum* eating throughout the study so that they could manipulate EI by themselves. The substantial difference in EI between the low EA and non-low EA groups may be attributed to this fact, with larger effect sizes of the difference than those in EEE.

Competitive runners likely have the drive to be thin [14], and the progressive trend of thinness was reported in a Japanese national high school competition [19] where the participants of the current study aimed to compete and win. Considerable leanness of our participants (mean %fat, 13.5 %) compared to female athletes in former studies on RMR (elite Scandinavian athletes, 20.0 % [8]; elite female sprinters, 20 % [36]; and ballet dancers, 17.3 % [7]), as well as the high frequency of amenorrhea, suggests that attitudes toward thin physiques, such as dietary restraint, are common in the team. It should be noted that participants with low EA had larger body mass, BMI, %fat, and fat mass than their counterparts. Interestingly, this feature was similarly observed among athletes with low EA in previous reports [8, 36, 37]. In contrast, running performances were better in the non-low EA participants than in the low EA participants. Such conspicuous images of “larger” or “fatter” bodies, in relation to inferior performance within the same team, are easily internalized and may be linked to the development of pressure to be thin [38]. These circumstances drive athletes to control weight by dietary restraint, which may underpin the development of low EA. Given the serious health consequences of low EA, particularly during adolescence, young competitive runners with low EA should be appropriately identified [1]. Although a reduction in RMR, which is a marker of energy deficiency [9], would equally represent low EA, the results of our investigation suggest that it does not necessarily accurately reflect the severity of low EA.

We observed that RMR reduced with the decline of EA toward the threshold value (30 kcal⋅kg^−1^ FFM⋅d^−1^) of low EA. However, further reduction in RMR was not observed along with continuous lowering of EA. This might suggest that RMR would be robustly sustained in low EA states among competitive athletes who regularly endure strenuous exercise training. Melin et al. [8] studied elite Scandinavian athletes engaged in endurance sports and obtained similar results; RMR (kcal⋅kg^−1^ FFM⋅d^−1^) of the athletes with the lowest EA (< 30 kcal⋅kg^−1^ FFM⋅d^−1^) was similar to those with optimal EA (≥ 45 kcal⋅kg^−1^ FFM⋅d^−1^) (*p* = 0.06). The analysis was performed by traditional group comparison using the operational cut-off values of EA. However, using a dichotomous or group comparison might lead to confusing results of their relationship, depending on which value is adopted for the analysis. In fact, we used EA = 30 kcal⋅kg^−1^ FFM⋅d^−1^ to distinguish low EA from non-low EA, with no significant difference observed in RMR. On the contrary, if we used EA = 45 kcal⋅kg^−1^ FFM⋅d^−1^ to differentiate optimal EA from non-optimal EA, RMR would be significantly lower in the non-optimal EA group than in the optimal EA group. This finding is consistent with the results of the study by Melin et al. [8].

Costa et al. [11] provided a correlation between EA and RMR in collegiate synchronized swimmers and showed that EA was significantly correlated with RMR indexed for body mass. However, FFM should be used to adjust RMR among athletes [9, 39]. When we calculated RMR indexed for FFM, there was no significant correlation of EA with RMR. Furthermore, RMR was lower than the predicted values, regardless of EA level, and there was no correlation between RMR ratio and EA, irrespective of the prediction formulae used. Conversely, Melin et al. [8] reported a positive correlation between RMR ratio and EA. They adopted Cunningham’s equation to predict RMR, which is considered appropriate for use in athletes, due to FFM being accounted for in the equation. [9, 39]. We tested the formulae specified for the race, age, and sex to calculate the predicted values as well as Cunningham’s equation. However, according to the correlation analysis, it seemed that RMR ratios provided by any formula could not appropriately reflect the decrease in EA. The limitation of using predicted values is that there is no universal standard equation that is appropriate for specific populations of athletes from different backgrounds [9]. Seeking appropriate equations is beyond the scope of this study and, above all, the discrepancies between the former and current studies regarding the relationship between RMR ratio and EA could be accounted for by the finding that a decrease in RMR was not proportional to a decrease in EA in our participants.

RMR is reduced by caloric restriction among sedentary individuals [10]. Such an adaptive response is similarly reported in adult athletes [40–43]. The present study indicated that RMR in teenage female athletes would reduce according to a decrease in EI. Although the association might have been due to an increased false-positive rate from multiple testing, the finding is consistent with those of previous studies. We expected that RMR would equally be suppressed with a decrease in EA; however, the association of RMR with EA was not linear; RMR seemed to reduce with a decline in EA only above the threshold of low EA, but not below it, where RMR was disproportionately higher than expected from the relationship above the threshold. The RMR of the six participants with low EA were the lowest (25.9 ± 1.7 kcal⋅kg^−1^ FFM⋅d^−1^) values reported by previous studies of adult female athletes (26.9–33.2 kcal⋅kg^−1^ FFM⋅d^−1^) [7, 8, 33, 36, 39]. Once RMR is suppressed to the lowest level for sustaining athletic activities, it may not be reduced further; however, EA can be further decreased.

A paradoxical increase in RMR despite energy deprivation has been reported. Koehler et al. [44] studied changes in the weight and RMR of sedentary normal-weight women by exercise training and caloric restriction. Most of the participants with a severe energy deficit (−1062 kcal⋅d^−1^) showed large weight loss; however, RMR remained unchanged. Zauner et al. [45] reported that starvation induced an increase in the RMR. Further, studies on chronically undernourished but apparently healthy participants demonstrated that RMR was not reduced, but rather higher than their well-nourished counterparts [46, 47]. A recent study of obese participants who maintained reduced body mass by weight-loss interventions substantiated these findings; long-term “weight-loss maintainers” did not necessarily show the adaptive suppression of RMR as expected [48].

Several factors can be considered for the disproportionally high RMR in participants with low EA. First, EA includes energy expenditure relevant to non-exercise activity and diet-induced thermogenesis, and larger decreases in these components may occur in participants with low EA to avert further suppression of RMR. Second, thermogenic responses by adrenaline infusion were not reduced in underfed conditions [49]. This may relate to elevated sympathetic activity and lipolysis in adipose tissue during exercise, even in chronic underfeeding [50]. Low EA participants might mobilize substrates from adipose tissue to a greater extent than participants with optimal EA during exercise and subsequently sustain RMR. Additionally, whole-body protein synthesis is similarly related to resting energy metabolism. Muscle protein synthesis is reduced by acute energy deprivation [51], except in chronically undernourished individuals [47]. Similarly, the reduction is negated by resistance exercise with FFM preservation [52]. While resistance exercise impact on RMR of adolescent female athletes with low EA has not been addressed, a protective effect of resistance exercise on RMR during caloric restriction has been described. Resistance training combined with diet decreased fat mass but preserved FFM and RMR in adult women [53] and middle-aged obese individuals [13] compared to diet only groups. Silva et al. [54] showed that competitive athletes could increase FFM while losing fat mass despite a negative energy balance. Additionally, we reported similar results for runners of the same team investigated in the present study [55]. Our participants regularly engaged in resistance training, which might prevent a substantial loss of skeletal muscle mass and potentiate metabolic activity at rest in low EA states. Further research is needed to define what mechanisms are responsible for the effect of low EA on resting energy metabolism.

To the best of our knowledge, this is the first research demonstrating that RMR would be sustained even in low EA states among competitive teenage girl runners. A major strength of this research is the use of a rigorous method to evaluate resting energy expenditure. A change of approximately 10 % could occur in measured RMR according to the condition of the participants [23]. The errors occur in relation to the thermic effect of food, physical movements after getting out of bed, transferring to a laboratory, and emotional stress when preparing for the test. Therefore, participants should ideally stay and sleep overnight at the laboratory just before the measurement early in the morning, with a long duration of abstinence from food and exercise [23]. However, the procedure is time-consuming and requires a specific facility, and former studies on EA in athletes reasonably measured RMR in less restrictive conditions [7, 8, 11]; subjects either arrived themselves or were transported to the laboratory on test day. All measurements of the current investigation were performed in full accordance with a traditional standard for the basal metabolic rate measurement [23], and we used a whole room calorimeter with high precision mass spectrometry to measure RMR. This allowed us to conduct analyses without these errors.

Another scientific merit of this study is that our research provides additional data on Asian athletes to the field of research on EA. This investigation will similarly provide new insight on low EA concerning resting energy metabolism. The non-linear relationship between EA and RMR draws attention to the use of a reduced RMR as a surrogate marker for low EA. Finally, the results were obtained from free-living athletes. We could conduct the measurements without an adverse effect on the daily routines of training and student life. Clinically, this would help generalize the results to athletes in real life.

Several limitations of the present study should be addressed. First, to seek homogeneity of participants and use a whole room calorimeter, we could not substantially increase the number of participants. Due to the small number of participants, the correlation analysis results between EA and RMR should be carefully interpreted. However, we would like to highlight runners with relatively high RMR despite extremely low EA, rather than determine the statistical significance by testing a null hypothesis, which can be determined by larger sample sizes. Second, a cross-sectional design was used for the study, and changes in body mass, body composition, or the long-term history of energy balance in the runners were not investigated. Hence, longitudinal observation is necessary to elucidate the chronic effect of low EA on RMR. Besides, we were not able to describe if our results were reflected by race- or age-specific effects. The low EA threshold [1] was based on studies conducted on adult Caucasians. More evidence is needed to determine if the cut-off value of low EA can be applied to Asian adolescent athletes. Even though they had lower EA than the threshold, they might be able to perform without substantial health risk. Finally, errors may have somewhat occurred when EI and EEE were assessed; however, it was impractical to use more accurate methods, such as a prepared diet or monitoring VO_2_ during training sessions, in free-living athletes. We used parent reports [56] and food photography [57] to confirm the food records of the participants to evaluate EI with greater accuracy. Additionally, we utilized running speed instead of HR to achieve a more accurate estimation of EEE during running training. HR continuously increased for more than 10 min of moderate- or high-intensity exercise, even if the intensity (e.g., running speed) was constant (cardiovascular drift) [58]. This would likely occur in higher intensity and longer duration exercises. Since running was the major component of EEE among the participants, and each training session was of high intensity and continued for several minutes (20 to 60 min), EEE would be overestimated if HR were used for estimation.

## Conclusions

This study showed that low EA is commonly observed among highly competitive and lean Asian girl runners, as reported in Caucasian adult female athletes. Some of them had extremely low RMR. However, the study results suggest that the RMR did not change with a decrease in EA among these girl runners, particularly when the EA of the runners was below the threshold of low EA. Furthermore, the degree of RMR suppression compared to the predicted value may not appropriately reflect the EA level. These findings might be related to race- or age-specific effects or protective effects of resistance training on RMR. Although further studies with experimentally induced low EA are necessary to confirm the findings, the identification of free-living teenage girl runners with low EA using RMR as a proxy for EA change should be performed cautiously, considering the above findings.

## Data Availability

The datasets generated and analyzed during the current study are not publicly available because the name of each subject can be identified by the height, weight, and performance records. However, they are available from the corresponding author on reasonable request.

## Abbreviations

EA: energy availability
EI: energy intake
EEE: exercise energy expenditure
RMR: resting metabolic rate
DXA: dual-energy X-ray absorptiometry
VO_2_: oxygen consumption
HR: heart rate
FFM: fat free mass
CV: coefficients of variation
J1: race, age, and sex-specific formulae to provide predicted RMR
J2: the equation developed for Japanese adult female athletes accounting FFM to provide predicted RMR
C: Cunningham’s equation
BMD: bone mineral density
BMD_TBLH_: bone mineral density of total body less head
